# Manual Dexterity Impairment in Patients with Complete Paraplegia: An Exploratory Study

**DOI:** 10.3390/brainsci16060566

**Published:** 2026-05-27

**Authors:** Chiara Pavese, Marta Mirando, Benedetta Cazzulani, Valeria Pingue, Antonio Nardone

**Affiliations:** 1 Department of Clinical-Surgical, Diagnostic and Pediatric Sciences, University of Pavia, 27100 Pavia, Italy; chiara.pavese@unipv.it (C.P.); marta.mirando@unipv.it (M.M.); valeria.pingue@unipv.it (V.P.); antonio.nardone@unipv.it (A.N.); 2 Istituti Clinici Scientifici Maugeri IRCCS, Centro Studi Attività Motorie (CSAM) and Neurorehabilitation and Spinal Units of Pavia Institute, 27100 Pavia, Italy; benedetta.cazzulani@icsmaugeri.it

**Keywords:** spinal cord injuries, paraplegia, rehabilitation, movement, upper extremity

## Abstract

**Highlights:**

**What are the main findings?**

• Individuals with complete paraplegia as a consequence of spinal cord injury show reduced manual dexterity during the Purdue Pegboard Test, despite clinically normal upper extremity strength and sensation.
• Reduced manual dexterity in paraplegia may hypothetically reflect altered spinal and supraspinal changes induced by the diminished ascending somatosensory input.
• Ageing negatively affects assembly performance of the Purdue Pegboard Test in individuals with complete paraplegia.

**What are the implications of the main findings?**

• Findings highlight the need to assess manual dexterity in individuals with paraplegia, even when clinical muscle strength and sensory examination are normal.
• Understanding altered dexterity may inform rehabilitation strategies targeting supralesional plasticity.

**Abstract:**

**Background**: Spinal cord injury (SCI) impairs sensorimotor function below the lesion and reshapes supralesional circuits, potentially influencing motor control above the injury. Although upper extremity strength and sensation are clinically normal in paraplegia, it is not known whether supralesional reorganization may produce subclinical alterations in the fine motor skills of the upper extremities. The aim of the study was to compare manual dexterity evaluated through the Purdue Pegboard Test between patients with SCI (PwSCI) and healthy subjects (HS). **Methods**: We recruited 18 PwSCI with complete paraplegia and 18 age- and sex-matched HS. Participants completed the four subtests of the Purdue Pegboard Test: dominant hand (1), non-dominant hand (2), bimanual (3), and assembly (4). For the first three subtests, a mixed 3 × 2 ANOVA (3 subtests × 2 groups) was performed, whereas for the fourth subtest, an independent samples *t*-test was performed. Spearman’s rho quantified correlations among subtests and with clinical findings. **Results**: PwSCI showed full upper extremity muscle strength and sensation. The mean scores of the four subtests were significantly lower in PwSCI than in HS. Unilateral and bimanual subtests correlated with each other in both groups; however, the bimanual subtest was not as well predicted by dominant hand alone, as PwSCI depended more on non-dominant ability. In PwSCI, the assembly subtest strongly depended on dominant, non-dominant, and bimanual scores, whereas this dependency was weaker in HS. The subtests were influenced by ageing in PwSCI. **Conclusions**: Despite upper extremity muscle strength and sensation being clinically normal, PwSCI showed impaired manual dexterity. This may reflect diminished ascending somatosensory input to supraspinal centres and plastic changes in supralesional motor pathways. These preliminary results open new rehabilitation perspectives for PwSCI.

## 1. Introduction

Spinal cord injury (SCI) is a catastrophic event leading to sensory and motor impairment below the level of the lesion. Depending on the level of the lesion, the impairment may be tetraplegia (weakness of all four limbs) or paraplegia (weakness of the lower limbs), the latter occurring when the lesion is below T1 [1]. In patients with spinal cord injury (PwSCI) with paraplegia, the clinical assessment of the upper extremity according to the International Standards for Neurological Classification of Spinal Cord Injury shows full muscle strength [2] and sensation [3,4]. The assessment takes into consideration, among others, the muscle strength and sensation of five key muscles and five key sensory points of both upper extremities, spanning from C5 to T1 spinal segments [3,4]. However, the finding of normal strength and sensation at these key muscles and points does not indicate that fine manipulation is also intact in PwSCI. In this context, it is known that, in humans, the cortical control of distal upper extremity movements, particularly deputed to fine manipulation, is different from that of the proximal extremity [5,6,7]. In addition, the motoneurons of the proximal upper extremity muscles are located in the medial and ventral columns of the spinal cord along several segments, whereas the motoneurons of the distal extremity muscles are located in the lateral columns of the spinal cord [8]. Finally, studies with transcranial magnetic stimulation suggest that the control of precision grip is complex, involving disynaptic or polysynaptic spinal pathways to the motoneurons innervating finger muscles in addition to monosynaptic corticomotoneuronal pathways [9].

In paraplegia, the central nervous system pathways above the lesion are deprived not only of the sensory input from the neural segments below the lesion, but also of the propriospinal circuits that bidirectionally connect the motoneurons deputed to the innervation of the upper and lower extremities (see [10], for a review). In addition, the brain of PwSCI is capable of extensive reorganization [11]. This can lead to alterations of the descending motor pathways, which also affect the spinal segments above the lesion [12,13]. As an example, in PwSCI, studies with functional Magnetic Resonance Imaging (fMRI) during finger and hand movements showed marked abnormalities in brain activation [14], partly due to cortical reorganization as a consequence of the loss of afferent input [15].

We hypothesized that, due to the above-cited reorganization of the spinal circuits spared by the lesion, malfunctions in fine motor control, such as those underlying manual dexterity, cannot be ruled out despite clinically normal motor and sensory evaluations of the upper extremity. The purpose of our study was therefore to evaluate the effect of SCI that resulted in paraplegia on manual dexterity. For this purpose, we compared the performance of fine manipulation between PwSCI with paraplegia and healthy subjects using a well-known standardized test of manipulation, the Purdue Pegboard Test, which has already been applied in other neurological diseases [16,17,18]. It revealed that PwSCI leading to paraplegia showed an impairment of manual dexterity that should be taken into consideration when performing rehabilitation to improve autonomy in activities of daily living.

## 2. Materials and Methods

### 2.1. Subjects

The study conformed to the standards established by the Declaration of Helsinki and was approved by the local ethics committee (approval number 2634 CE). Before entering the study, all patients were informed about the procedures and provided written informed consent to participate.

We recruited patients with spinal cord injury (PwSCI) leading to complete paraplegia who were admitted to the Spinal Cord Injury Unit of Istituti Clinici Scientifici Maugeri, Pavia, Italy, between April 2022 and February 2025, as well as an equal number of age- and sex-matched healthy subjects (HS).

Inclusion criteria for the PwSCI were age between 18 and 74 years; complete spinal cord injury (American Spinal Injury Association Impairment Scale, AIS, grade A) [3,4] of any cause; subacute or chronic phase; capable of understanding instructions; neurological level of injury (NLI) below T1, with full strength and sensation up to T1; and capacity to sit in a wheelchair for half an hour. Exclusion criteria were severe visual deficits; cognitive impairment or other concomitant neurological and psychiatric diseases; and reduced upper extremity function due to conditions of various etiologies (e.g., neurological, such as stroke or brachial plexus injury, or orthopedic). HS were recruited from the relatives or clinical staff who were unfamiliar with the testing procedures using 1:1 matching criteria and an age range of ±5 years.

### 2.2. Clinical Evaluations

PwSCI were evaluated with the International Standards for Neurological Classification of Spinal Cord Injury (ISNCSCI) to assess eligibility [3,4]. The ISNCSCI is the gold-standard evaluation introduced by the American Spinal Injury Association and validated for patients with traumatic and non-traumatic SCI [3,4]. The ISNCSCI defines the NLI and the AIS grade. NLI is defined as the lower spinal cord segment with normal sensation and antigravity muscle strength, with full sensory and motor function above. The AIS grade indicates the degree of completeness and expresses the severity of SCI, ranging from A (complete impairment) to E (normal function).

For each patient, we noted the drugs taken on the day of the test, which could potentially have an interaction with the performance: muscle relaxants, antidepressants, antiepileptics, and anxiolytics.

### 2.3. Manual Dexterity Evaluation

All evaluations were performed in the same setting at a standardized table. PwSCI were assessed while seated in their own wheelchairs; HS were assessed while seated on a standard chair. Manual dexterity was evaluated through the Purdue Pegboard Test [19,20,21]. The board shows two parallel rows of 25 holes each. The test consists of four subtests. In three of them, both PwSCI and HS were required to place as many pins as possible in the holes within 30 s. One subtest was performed with the dominant hand, another with the non-dominant hand, and one more with both hands simultaneously. In the fourth subtest, the subjects used both hands for 60 s to construct as many assemblies as possible formed by single parts consisting of a pin, two washers, and a collar. The first and second scores represent the number of pins inserted with the dominant and non-dominant hands, respectively. The third score reflects the number of pin pairs inserted, while the fourth indicates the total number of single parts used to construct full or partial assemblies. The sequence of the subtests was randomized between subjects. Before starting the trial, the experimenter demonstrated the movement and then allowed participants to practice the trial. Participants were instructed on when to start and end each subtest via a stopwatch [22,23]. Each subtest was then performed once to provide four separate scores.

### 2.4. Sample Size Calculation

The sample size calculation was based on the score of subtest 4. Sample size was determined for a two-sample, two-sided comparison of means with 1:1 allocation (α = 0.05, power = 80%). From our preliminary data (PwSCI 23.9 ± 6.7; HS 31.4 ± 7.1), the pooled standard deviation was 6.90, and we therefore considered a minimum significant difference of 8 points.

For the calculation, we considered a test power of 80% and a type I error equal to 0.05. The following formula was applied:

$${n>2[\left(\frac{\left(\frac{z\alpha }{2}\;+\;z\beta \right)}{\delta }\right)\sigma ]}^{2}$$

where z α/2 = 1.96; zβ = 0.842, for a power of 80%; σ (standard deviation in the PwSCI sample) = 6.90; and δ (prespecified minimal significant difference) = 8. Based on this, the resulting minimum number of subjects for each group was 12.

### 2.5. Statistical Analysis

Data were reported as mean and standard deviation. Chi-squared test was applied to assess the association in contingency tables. A mixed 3 × 2 ANOVA (3 subtests × 2 groups) was applied to assess the difference between the three different scores in the two groups. An independent samples Student’s *t*-test was applied to assess differences in quantitative variables between the two groups. Spearman’s rho correlation coefficients were calculated to quantify the coupling between Purdue Pegboard scores in the four subtests, as well as the relationship between scores and clinical findings. The rho values were normally transformed through Fisher’s Z transformation, and any significant differences between pairs of rho were assessed with the Z-test. Statistical significance was set at *p* < 0.05. We used IBM SPSS Statistics v.29 and Microsoft Excel for the statistical analysis and for the statistical power assessment.

## 3. Results

### 3.1. Subjects

We enrolled 18 PwSCI with complete injury (AIS A; injury level T2–L1; mean age 47.3 ± 13.8 years; 13 men, 72.2% of total) and 18 age-matched HS (mean age 46.9 ± 10.6 years; 11 men, 61.1%). Neither age nor sex distribution differed between groups (*t*-test, *p* = 0.91; Cohen’s d = 0.04, and chi-squared, *p* = 0.48, Cramer’s V = 0.12). SCI etiology was traumatic in 13 cases (72.2%), vascular in two (11.1%), and post-surgical in three (16.7%). The mean time from SCI to Purdue Pegboard Test evaluation was 6.9 ± 7.9 years. All subjects were right-handed. No clinically evident motor or sensory deficits of the upper extremity were present in PwSCI according to the ISNCSCI. Nine (50%) of patients were taking muscle relaxants, two (11%) antidepressants, six (33%) antiepileptics, and five (28%) anxiolytics. Demographic and clinical characteristics are summarized in Table 1.

### 3.2. Manual Dexterity in PwSCI and HS

**Figure 1:**
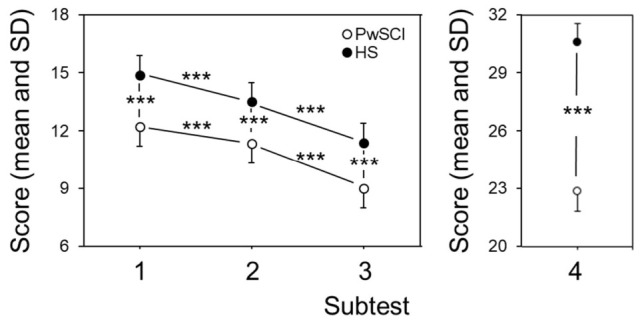
Scores of the subtests derived from the Purdue Pegboard Test administered to patients with SCI (PwSCI, open symbols) and healthy subjects (HS, filled symbols). Left panel: subtest 1 = dominant hand; subtest 2 = non-dominant hand; and subtest 3 = bimanual. Right panel: subtest 4 = assembly. Scores are reported as mean and standard deviation (SD) of the number of pegs for subtests 1 and 2; of the number of peg pairs for subtest 3; and of the number of single parts used to construct full or partial assemblies for subtest 4. Asterisks indicate significant differences between scores; ***, *p* < 0.001.

All subjects were able to perform the four subtests without any problems. As shown in Figure 1 and Table 2, the scores obtained in each of the four subtests showed a similar behaviour in the two subject groups.

Considering only the scores of subtests 1–3 (Figure 1, left panel), since these were comparable as they were all performed within 30 s, there was homogeneity of variances and covariances (*p* > 0.05), as assessed by Levene’s test of homogeneity of variances and Box’s M test, respectively. Mauchly’s test of sphericity indicated that the assumption of sphericity was met for the two-way interaction with χ^2^(2) = 1.136, *p* = 0.57. There was no statistically significant interaction between the subject groups and the scores according to mixed ANOVA 2 × 3: F (2, 68) = 0.38, *p* = 0.69, partial η^2^ = 0.011, observed power = 0.109. The main effect of the three subtests showed a statistically significant difference among the three scores as F (2, 68) = 70.72, *p* < 0.001, partial η^2^ = 0.675, observed power = 0.999. The subsequent pairwise comparison showed statistical differences (*p* < 0.001) in all the comparisons. The main effect of group showed that there was also a statistically significant difference in mean scores between subject groups as F (1, 34) = 20.11, *p* < 0.001, partial η^2^ = 0.372, *p* < 0.001.

Regarding subtest 4 (Figure 1, right panel), an independent samples *t*-test was performed to determine if there were differences between PwSCI and HS groups. The score was more elevated in HS than PwSCI, with a statistically significant difference as t (34) = 4.287, *p* < 0.001.

### 3.3. Correlations Between Subtest Performances in PwSCI and HS

To assess whether the poorer performance in PwSCI at the assembly subtest was related to an impairment in the performance with either the dominant or non-dominant hand, or with both, we correlated the scores obtained from the various pairs of subtests in the two subject groups. The results are shown in Table 3 and in the following figures.

**Figure 2:**
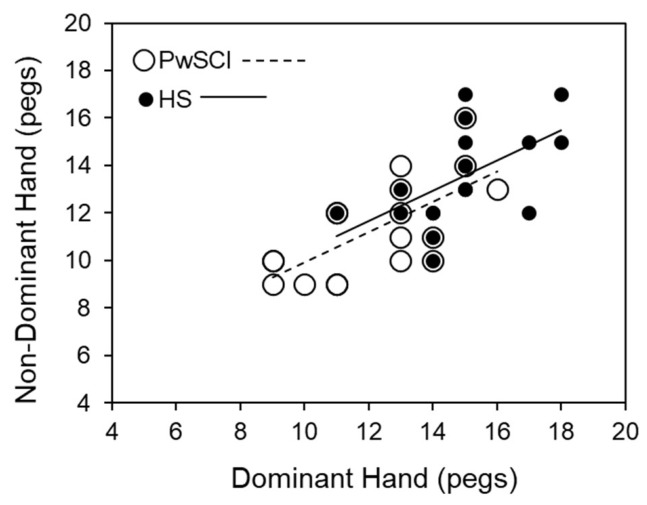
Correlations between the scores (number of pegs) of subtest 2 (non-dominant hand) and subtest 1 (dominant hand) in patients with SCI (PwSCI, open symbols, shaded line) and healthy subjects (HS, filled symbols, continuous line). The open symbols are larger than the filled ones to make it easier to see any superimposed score values.

Figure 2 shows a significant (rho = 0.67, *p* < 0.005) positive correlation between non-dominant and dominant hand subtests (score 2 vs. 1), in both PwSCI and HS (Table 3), though the rho value is lower than 1, suggesting that the dominant hand performed better. In addition, the correlation coefficients were not significantly different (Z-test, *p* = 0.94) between the two subject groups, indicating that the performances of the two hands were associated in both groups despite the lower mean score in PwSCI (see Figure 1 and Table 2).

**Figure 3:**
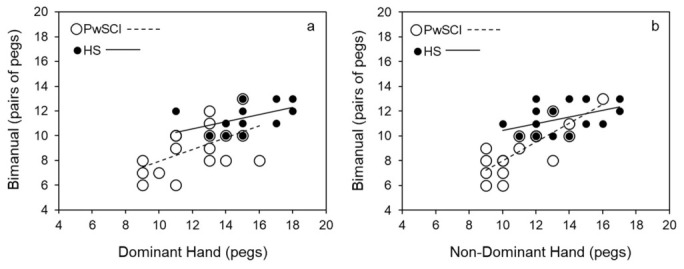
Correlations between (**a**) the scores of subtest 3 (bimanual) and subtest 1 (dominant), and (**b**) the scores of subtest 3 and subtest 2 (non-dominant) in patients with SCI (PwSCI, open symbols, shaded line) and healthy subjects (HS, filled symbols, continuous line). Scores correspond to the number of pegs for subtests 1 and 2, and to the number of peg pairs for subtest 3. The open symbols are larger than the filled ones to make it easier to see any superimposed score values.

Figure 3a,b show the relationship between the scores of subtest 3 (bimanual) and subtest 1 (dominant hand) and between the scores of subtest 3 and subtest 2 (non-dominant hand) in the two subject groups, respectively. Table 3 shows that, in both groups, the correlation coefficients of the scores of subtests 3 vs. 1 were positive (rho = 0.53, *p* < 0.05) in both PwSCI and HS and not significantly different (Z test, *p* = 0.99), suggesting that the bimanual subtest was dependent on the performance of the dominant hand in both PwSCI and HS. On the contrary, in the case of subtests 3 vs. 2, the correlation coefficient was strongly significant (rho = 0.79, *p* < 0.001) in PwSCI but not significantly so (rho = 0.43, *p* = 0.08) in HS. Therefore, the bimanual subtest was roughly independent of the performance of the non-dominant hand in HS, at variance with PwSCI. However, the correlation coefficients between subtests 3 and 2 were not significantly different in PwSCI and HS (Z-test, *p* = 0.11).

**Figure 4:**
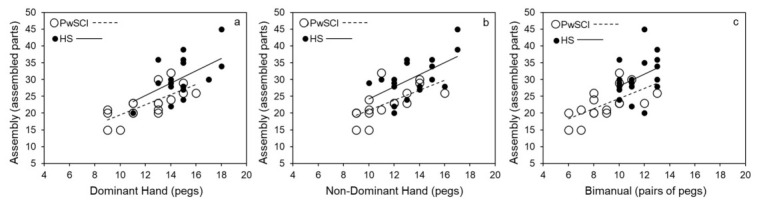
Correlations between the scores of subtest 4 (assembly) and (**a**) subtest 1 (dominant), (**b**) subtest 2 (non-dominant), and (**c**) subtest 3 (bimanual) in patients with SCI (PwSCI, open symbols, shaded line) and healthy subjects (HS, filled symbols, continuous line). Scores correspond to the number of pegs for subtests 1 and 2, to the number of peg pairs for subtest 3, and to the number of parts comprising each assembly for subtest 4. The open symbols are larger than the filled ones to make it easier to see any superimposed score values.

Figure 4a–c show the correlations between the scores of subtest 4 (assembly) and subtest 1 (dominant), subtest 2 (non-dominant), and subtest 3 (bimanual) in the two subject groups. In PwSCI, the slope of the correlations turned out to be significant in all three pairs of subtests (rho = 0.78, 0.79, 0.70; *p* < 0.001, for all comparisons) (Table 3). On the contrary, in HS, no significant correlations were found in any pair of subtests (rho = 0.41, 0.46, 0.37). However, the three pairs of correlation coefficients were not significantly different between PwSCI and HS (Z-test, *p* = 0.10, 0.12, 0.20).

### 3.4. Correlations Between Subtest Performances and Clinical Findings in PwSCI and HS

**Figure 5:**
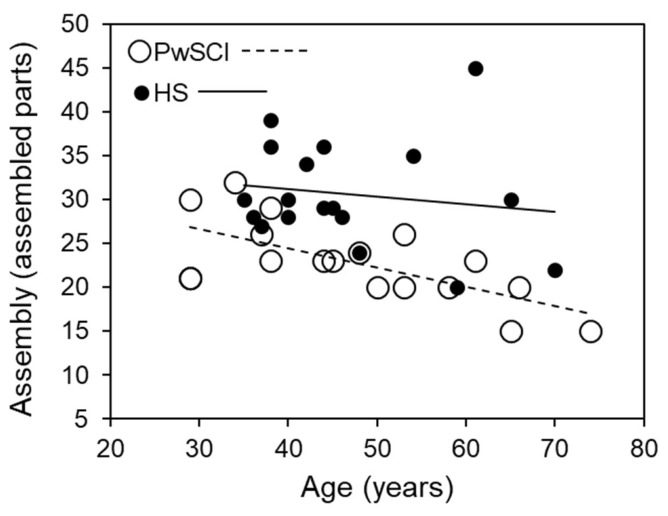
Correlations between the scores of the subtest 4 (assembly) and age in patients with SCI (PwSCI, open symbols, shaded line) and healthy subjects (HS, filled symbols, continuous line). Scores correspond to the number of parts composing each assembly. The open symbols are larger than the filled ones to make it easier to see any superimposed score values.

We sought correlations between the scores obtained in each of the four subtests and the subjects’ age, level of injury, and time since injury. No correlation was found in any subject group apart from age. In PwSCI, except for subtest 1 (dominant: rho = −0.30, *p* = 0,22), there was a significant correlation between age and subtest scores—specifically, subtest 2 (non-dominant: rho = −0.47, *p* = 0.047), subtest 3 (bimanual: rho = −0.48, *p* = 0.044), and subtest 4 (assembly: rho = −0.65, *p* = 0.004; see Figure 5)—indicating a progressively worsening performance with ageing. On the other hand, in HS, correlations with age were always negligible except for the score of subtest 2 (non-dominant: rho = −0.498, *p* = 0.035). No significant difference for any correlation between PwSCI and HS was present (Z-test, *p* = 0.94, 0.93, 0.29, and 0.11 for subtest 1, 2, 3, and 4, respectively).

## 4. Discussion

The results show that patients with SCI (PwSCI) with complete paraplegia below T1, despite the absence of clinically evident motor and sensory signs in the upper extremities, exhibit impairments in manual dexterity across all four subtests of the Purdue Pegboard Test. In both PwSCI and HS, a positive correlation existed between the performance of the dominant and non-dominant hand, but the dominant hand generally performed better than the non-dominant. In addition, in both HS and PwSCI, the performance of the bimanual subtest was fairly correlated with the performance of the dominant hand. On the contrary, in PwSCI, the bimanual subtest was strongly correlated with the performance of the non-dominant hand. Further, in PwSCI, the assembly subtest was strongly dependent on the performance of either hand, as well as of the bimanual subtest. Finally, no correlations were found between the subtests and the clinical findings, except for a negative correlation between subtest performance and age, particularly in PwSCI.

### 4.1. Impairment of Manual Dexterity in PwSCI

Our sample of patients with paraplegia did not show any clinically evident change in muscle strength and sensitivity according to the ISNCSCI, a well-known finding in these patients for many years [3,4,24]. Since our preliminary study lacks neuroimaging and/or neurophysiological data, we can only speculate about the causes of the impairment in manual dexterity in PwSCI with complete paraplegia. On the one hand, we feel that reduced fine dexterity can hardly be interpreted because of a reduction in simple reaction times of the upper extremities. In fact, it has been shown that the reaction times of PwSCI with complete paraplegia in a task of key pressing are not delayed with respect to HS [25]. On the other hand, it is known that several neurophysiological and neuroimaging changes occur above the lesion because of disruption of somatosensory afferent pathways, and of plastic changes in supralesional descending motor pathways [12,13]. These phenomena may play a prevalent role in affecting manual dexterity rather than the control of the proximal upper extremity of PwSCI due to the different organization of cortical control and spinal circuits subserving proximal with regard to distal upper extremity movements in humans (see [5], for a review). It has also been shown that, in patients with paraplegia, during voluntary hand movements, brain activation is abnormal. For example, it has been reported that the reduction in sensory input from the spinal cord to the supraspinal centres may lead to an increased activation of the thalamus and cerebellum, both activating to a larger extent the somatosensory cortex during voluntary movements of the hand [11,26]. In addition, even in lesions classified as clinically complete, preserved subclinical sensory connections and altered afferent integration have been documented. This supports the idea that the quality, not merely the presence or absence, of sensory inflow may change after SCI [27] and eventually influence fine manipulation.

A role in the impairment of dexterity in PwSCI might be connected not only to changes in afferent processing and supraspinal reorganization of motor pathways but also to the loss of axons in long propriospinal neurons connecting lumbar and cervical neuronal circuits (see [10,28]), which can be interrupted by spinal injury. However, according to results obtained by [29], it is unlikely that the impairment in manual dexterity depends on the injury disrupting the coupling of cervical and thoraco-lumbar propriospinal systems. In fact, during skilled hand movements while seated, the direct cortico–motoneuronal excitation of cervical spinal circuits predominates whilst the coupling of cervical and thoraco–lumbar propriospinal neuronal system is inhibited [30].

### 4.2. Correlations Between Subtest Performances in PwSCI and HS

The observation that, in both PwSCI and HS, a positive correlation existed between the performance of the dominant and non-dominant hand, but that the dominant hand performed generally better than the non-dominant comes as no surprise [31]. More interestingly, we found that, only in PwSCI, the bimanual subtest was strongly correlated with the performance of the non-dominant hand. This group difference suggests that PwSCI may solve the bimanual condition in a more serial, hand-limited manner (i.e., the slower/less efficient hand restricts the overall performance), whereas HS may benefit from more efficient inter-hand coordination and/or greater flexibility in their strategies, which makes the bimanual subtest less “dominated” by a single-extremity bottleneck. Finally, only in PwSCI, the assembly subtest was strongly dependent on the performance of either hand, as well as with the bimanual subtest. This is consistent with the assembly subtest placing greater demands on sequencing, the rapid switching between the reach-grasp-place phases, and the fine bimanual stabilization of the elements to be assembled, thereby amplifying the impact of subtle deficits in feedback control and coordination [32].

### 4.3. Correlations Between Clinical Findings and Subtest Performances

Our sample comprised both subacute and chronic patients, which could have led to an underestimation of patients’ impairment in manual dexterity. However, according to fMRI study, it has been shown that, in PwSCI, a decrease in spinal cord area, white matter volume of the corticospinal tracts, and grey matter volume in the sensorimotor cortex occurs as early as 40 days after the injury [33]. These changes have been interpreted as retrograde degeneration of myelinated axons [34], and it is known that the degree of spinal [35] and brain atrophy correlates with the severity of motor and sensory impairment [36]. In this context, the presence of both subacute and chronic participants may increase variability because neuroplastic and degenerative processes evolve dynamically over time. However, we did not find any association between time since injury and manual dexterity, and this is possibly due to the fact that the effects of time since injury may be non-linear and not captured by simple correlation analyses, particularly in small samples [37], as in our study.

The worst performance of elderly PwSCI than HS on most subtests is in line with previous findings; it is known that, despite the same strength and sensitivity, elderly patients have greater difficulty in translating their neurological potential into functional tasks [38]. In addition, the differences in manual dexterity between PwSCI and HS can hardly be ascribed to differences in finger size, being greater in men than women [39], or in age [40], since the number of men and women and their ages were comparable in both subject groups. Finally, the results obtained in HS were superimposable to those of the literature [41,42], suggesting that the differences with PwSCI were not connected to a sampling bias of HS.

### 4.4. Strengths, Limitations of the Study, and Future Research Directions

The novelty of our study lies in the demonstration of a previously undetected subclinical alteration in manual dexterity in paraplegic subjects. The strength of the study resides in the possibility of assessing these alterations in manual dexterity, using a functional outcome measure commonly employed in clinical practice and rehabilitation. For example, the Purdue Pegboard Test has been used to assess the effect of passive somatosensory stimulation on upper extremity function in patients with chronic stroke [16], and to assess fine motor function of the hands in patients with cerebellar stroke [18]. Interestingly, both patients with chronic stroke [16] and those with cerebellar ataxia [18] were much more impaired than our patient group in both the dominant and non-dominant extremities, the only subtests assessed in these studies. The less severe impairment in PwSCI than in the above neurological diseases underlines the fact that our patients feature only a subclinical impairment of manual dexterity. Therefore, it comes as no surprise that this impairment cannot be put into evidence simply by the ISNCSCI evaluation of the upper extremity performed during physical examination.

Confirmatory studies conducted on larger samples of PwSCI might suggest adapting normative scores of the Purdue Pegboard Test [21] to these patients as well, even including those with paraplegia. In addition, examining the manual dexterity of PwSCI and comparing it to normative values will contribute to the early detection of impairments due to overuse injuries, and to target manual dexterity in rehabilitation programmes, also in patients with complete paraplegia. This is particularly important in several activities of daily living, such as self-catheterization or vocational tasks, when optimal manual dexterity assumes utmost importance.

Several limitations stem from potential confounding factors that are difficult to fully quantify or control in studies involving PwSCI. Prolonged hospitalization or extended periods of inpatient rehabilitation may influence performance on the pegboard through deconditioning, fatigue, changes in daily activity levels, and reduced exposure to real-world manual tasks [43]. In addition, the use of medications—common in PwSCI for neuropathic pain, spasticity, anxiety, and depression—may affect attention, reaction time, psychomotor speed, and perceived effort, thereby altering pegboard outcomes independently of true manual dexterity [44]. More broadly, secondary complications frequently associated with SCI (e.g., spasticity, sensory deficits, autonomic symptoms, upper extremity overuse syndromes, sleep disturbances, and mood changes) can introduce variability in task execution. Secondary complications may mask or mimic functional change, affect attention, reaction time, psychomotor speed, and perceived effort, thereby altering pegboard outcomes independently of true manual dexterity [44]. Pegboard performance can be influenced by contextual factors such as assistive supports and day-to-day fluctuations in pain and fatigue, which may not be captured by a single assessment session [45,46]. Regarding factors such as seating and trunk stability, all PwSCI were seated in their usual wheelchair during the test. Although this introduced an experimental variability in the test condition between PwSCI and HS, it allowed a collection of data in an ecological context. These limitations should be considered when interpreting pegboard-based comparisons and longitudinal changes.

Our study includes patients with complete paraplegia of different etiology, but this aspect should not influence our results since recent studies suggest comparable outcomes in subjects with traumatic and non-traumatic SCI [47]. Other aspects of our inclusion criteria (NLI or time since injury) may be considered a source of variability, although our analyses do not show an influence of these aspects on manual dexterity. Further, the small sample size, although supported by an a priori calculation, should be considered as a limitation of the study. Indeed, the sample size is adequate for the primary analyses, but the sample could be underrepresented for other secondary sub-analyses. Finally, neuroimaging, neurophysiological, or kinematic correlates were not considered in this preliminary study and should be considered in the design of future research.

## 5. Conclusions

The Purdue Pegboard Test has been used as an index of fine dexterity in neurological and rehabilitation contexts. Our results show that patients with complete paraplegia also exhibit a deficit in manual dexterity, and that in these patients, the subtests correlate negatively with ageing. Our preliminary evidence warrants further study on a larger sample of patients with paraplegia to obtain functional data that can serve as a basis for adapting future rehabilitation protocols.

## Figures and Tables

**Table 1:** Individual demographic and clinical characteristics of patients with complete SCI. Sex, age, etiology, level of injury, and years since injury are reported for each participant.

Patient	Sex	Age (Years)	Etiology	NLI	Time Since Injury (Years)
1	M	29	Trauma	T7	6.0
2	M	45	Trauma	L1	0.1
3	W	34	Trauma	T4	19.4
4	M	74	Spinal stroke	T10	0.4
5	M	38	Trauma	T12	13.5
6	W	37	Trauma	T5	19.6
7	M	53	Trauma	T9	8.2
8	W	48	Trauma	T12	0.2
9	M	29	Trauma	T7	4.5
10	M	61	Trauma	T12	14.2
11	M	53	Post-surgery	T7	0.2
12	M	50	Trauma	T9	0.4
13	W	44	Post-surgery	T8	0.3
14	M	65	Trauma	T4	15.1
15	M	58	Post-surgery	T7	20.1
16	W	38	Spinal stroke	T2	0.5
17	M	29	Trauma	L1	1.0
18	M	66	Trauma	T10	0.1

M, man; W, woman; NLI, neurological level of injury.

**Table 2:** Purdue Pegboard performance in PwSCI and HS: mean scores (±standard deviation) for each subtest.

Subtest	PwSCI Score	HS Score
Dominant	12.2 ± 2.2	14.9 ± 1.8
Non-dominant	11.3 ± 2.1	13.5 ± 2.0
Bimanual	9.0 ± 1.9	11.4 ± 1.2
Assembly	22.8 ± 4.6	30.6 ± 6.1

PwSCI, patients with spinal cord injury; HS, healthy subjects.

**Table 3:** Spearman’s rho correlation coefficients between the scores obtained during the different subtests in PwSCI and HS, their *p*-values, and the *p*-values of the difference in the correlation coefficients between PwSCI and HS.

Subtest	PwSCI rho	*p*-Value	HS rho	*p*-Value	*p*-Value rho PwSCI vs. HS
Non-dominant vs. Dominant	0.67	**0.002**	0.67	**0.003**	0.94
Bimanual vs. Dominant	0.53	**0.025**	0.53	**0.025**	0.99
Bimanual vs. Non-dominant	0.79	**<0.001**	0.43	0.08	0.11
Assembly vs. Dominant	0.78	**<0.001**	0.41	0.09	0.10
Assembly vs. Non-dominant	0.79	**<0.001**	0.46	0.06	0.12
Assembly vs. Bimanual	0.70	**0.001**	0.37	0.13	0.20

PwSCI, patients with SCI; HS, healthy subjects. Significant differences are in bold.

## Data Availability

The mean data presented in this study are available on request from the corresponding author. The dataset is not publicly available since the authors intend to use the dataset for future research and analyses.

## Abbreviations

The following abbreviations are used in this manuscript:

AIS	American Spinal Injury Association Impairment Scale
ANOVA	Analysis of Variance
CE	Ethics Committee
fMRI	Functional Magnetic Resonance Imaging
HS	Healthy Subjects
ISNCSCI	International Standards for Neurological Classification of Spinal Cord Injury
NLI	Neurological Level of Injury
PwSCI	Patients with Spinal Cord Injury
SCI	Spinal Cord Injury
SD	Standard Deviation
SPSS	Statistical Package for the Social Sciences
